# Unraveling Dandy‐Walker Malformation: A Comprehensive Literature Review and Case Insight

**DOI:** 10.1002/ccr3.70356

**Published:** 2025-03-24

**Authors:** Mohammad Bdair, Fathi Milhem, Zaid Sawaftah, Husam Hamshary, Hamza A. Abdul‐Hafez, Moath Hattab, Noor Nabresi, Omar Naseef

**Affiliations:** ^1^ Department of Medicine An Najah National University Nablus Palestine; ^2^ Pediatric Department Dr. Thabet Thabet Hospital Tulkarm Palestine; ^3^ Department of Radiology An Najah National University Hospital Nablus Palestine

**Keywords:** cerebellar vermis hypoplasia, congenital malformation, Dandy‐Walker spectrum disorders, hydrocephalus, multidisciplinary care, ventriculoperitoneal shunt

## Abstract

Dandy‐Walker spectrum disorder (DWSD) is a congenital abnormality of the brain, consisting typically of partial or complete hypoplasia of the cerebellar vermis, cystic dilation of the fourth ventricle, and enlargement of the posterior fossa. Approximately 1 in 10,000 to 30,000 live births is affected by this typically early‐onset neurological condition, which commonly presents with hydrocephalus, irritability, and poor motor coordination. Usually, the diagnosis is confirmed by anatomic features typical of computed tomography or magnetic resonance imaging (MRI). Management has been largely hydrocephalus‐oriented, usually by VP shunting and multidisciplinary follow‐up for neurological and developmental improvement in the long run. Herein is reported a case with a 5‐month‐old male presenting with DWSD, which has been documented in this paper along with his clinical presentation, imaging findings, and, most importantly, the response to the cerebrospinal fluid (CSF) diversion following the shunt. This case highlights the importance of early intervention, early comprehensive imaging, and a multidisciplinary approach, including genetic counseling, in optimizing the quality of life and managing complex developmental needs associated with DWSD.

**Summary**

- Dandy‐Walker spectrum disorders are rare congenital malformations requiring early diagnosis and individualized, multidisciplinary care.
- Prompt intervention, particularly for hydrocephalus, significantly impacts prognosis, while ongoing developmental and genetic counseling support families in managing this complex condition and improving long‐term quality of life.

## 1 Introduction

Dandy‐Walker spectrum disorders (DWSD) represent a continuum of congenital posterior fossa malformations characterized by varying degrees of cerebellar vermis hypoplasia or agenesis, cystic dilation of the fourth ventricle, and enlargement of the posterior fossa [1, 2]. Although traditionally referred to as “Dandy‐Walker syndrome,” contemporary literature recognizes that these anomalies exist on a spectrum rather than as a single categorical entity. DWSD affect approximately 1 in 10,000 to 30,000 live births and have a slightly higher prevalence in females [2].

Key symptoms in newborns include hydrocephalus (present in 80% of Dandy‐Walker malformation (DWM) cases), irritability, seizures, vomiting, poor coordination, and hypotonia [3, 4]. Diagnosis is confirmed by imaging such as CT scan, magnetic resonance imaging (MRI), and perinatal ultrasound, typically showing characteristic DWSD features [5].

The management of DWSD primarily focuses on treating hydrocephalus, usually by placing a ventriculoperitoneal (VP) shunt to drain excess cerebrospinal fluid (CSF) [3]. Early intervention and continuous monitoring are essential to improving the long‐term quality of life for such cases [6].

In this case report, we present a newborn patient with DWSD marked by a complex clinical course, along with a comprehensive literature review. This case highlights the unique diagnostic and management challenges for this significantly rare condition in the neonatal period.

## 2 Case Presentation

A 5‐month‐old male infant was delivered at term via cesarean section to a healthy mother. The family history was negative for congenital anomalies, neurological disorders, or genetic syndromes, which provided valuable context for genetic counseling and assessment of hereditary predispositions. The pregnancy was largely uneventful until a routine ultrasound at 19 weeks of gestation revealed cystic dilation of the fourth ventricle, borderline ventriculomegaly (9 mm bilaterally), and absence of the cerebellar vermis. These findings raised concerns about a potential congenital anomaly, prompting additional imaging and follow‐up with a pediatric neurologist after birth.

At delivery, the infant weighed 3.3 kg but presented with a weak cry and signs of respiratory distress. Apgar scores were 7 at 1 min and 8 at 5 min. Given the respiratory difficulties and the previously identified congenital abnormalities, the infant was immediately admitted to the neonatal intensive care unit (NICU) for further evaluation and management.

On physical examination, the infant's head circumference exceeded the 95th percentile, and the anterior fontanelle measured approximately four fingers in width, suggestive of macrocephaly. The infant exhibited tachypnea with mild subcostal retractions. Neurological examination revealed generalized hypotonia, a weak Moro reflex, diminished sucking reflex, and recurrent hiccups. Occasional suspected seizures were also observed. Cardiovascular examination at birth was unremarkable; however, a mild systolic murmur was detected during subsequent assessments. The infant was placed on 2 L/min of nasal cannula oxygen to maintain a SpO_2_ of ≥ 92%, and nasogastric (NG) feeding was initiated to support nutritional needs.

## 3 Method (Differential Diagnosis, Investigation, Management)

**FIGURE 1:**
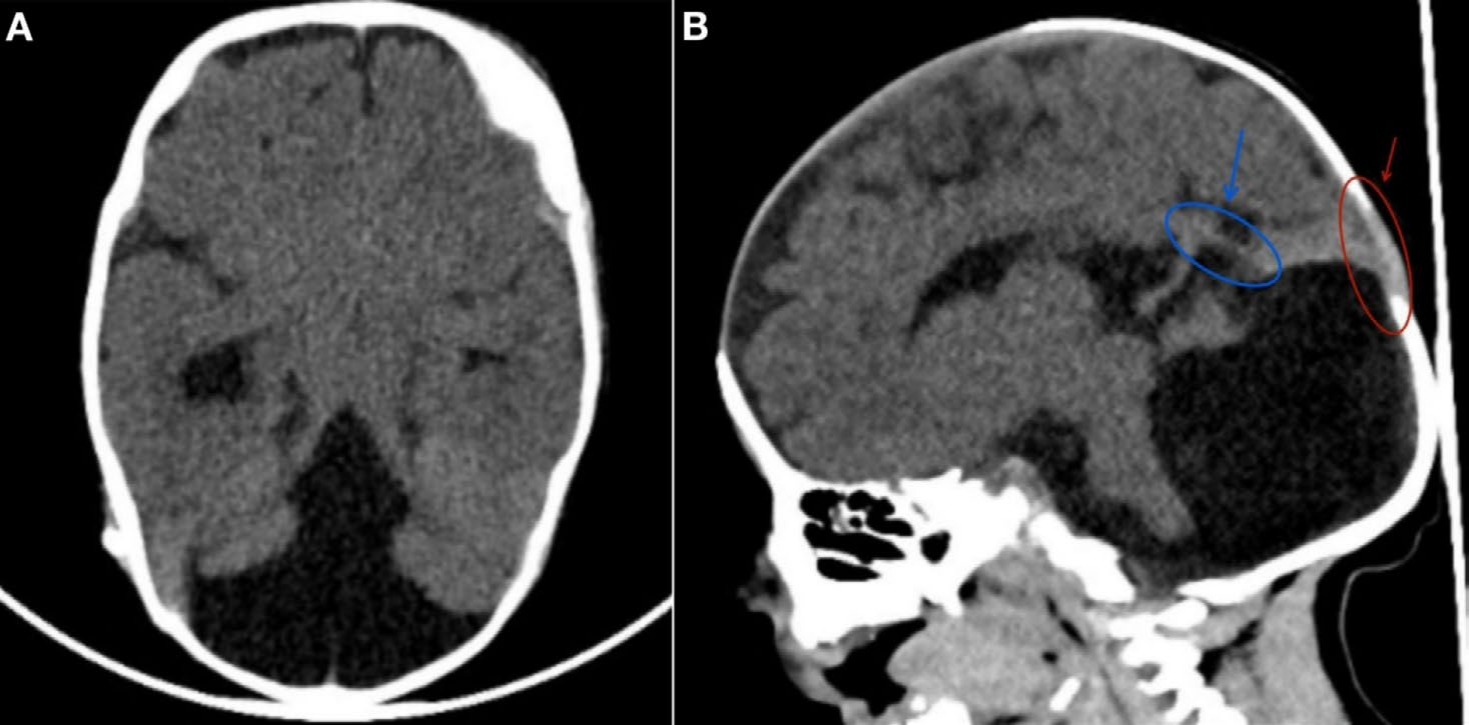
CT Scan of the Brain. (A) The transverse section reveals hypoplasia of the vermis. (B) The sagittal section demonstrates a dilated fourth ventricle that communicates with a mega cisterna magna. Additionally, the straight sinus and torcula (highlighted in blue) are positioned superior to the lambdoid suture (highlighted in red).

A comprehensive laboratory panel, including a full septic workup and serial labs, was performed, with unremarkable results (Table 1). Cardiac and renal evaluations identified a small muscular ventricular septal defect (VSD) with a left‐to‐right shunt and a patent foramen ovale (PFO), both of which were hemodynamically insignificant. Renal ultrasound findings were normal. A non‐contrast brain CT confirmed the prenatal findings, revealing cystic dilation of the posterior fossa, absence of the cerebellar vermis, and enlargement of the fourth ventricle. The straight sinus and torcula were positioned superior to the lambdoid suture, all consistent with DWM (Figure 1). Additionally, the CT scan demonstrated moderate dilation of the right lateral ventricle without signs of transependymal CSF permeation. No evidence of intracranial hemorrhage, midline shift, or other significant abnormalities was noted, and white‐gray matter differentiation appeared normal (Figure 1).

**TABLE 1:** Laboratory findings over multiple admissions show trends in blood sugar, complete blood count, inflammatory markers, liver enzymes, renal function, electrolytes, and cerebrospinal fluid analysis, highlighting the patient's metabolic and hematologic status across the clinical course.

	unit	Day 1	Day 2	Day 6	Day 9	5 months
Blood group & Rh Type		A+	—	—	—	—
Random blood sugar	mg/dL	51	—	—	—	82
**Complete blood count**						
White blood cells	K/μL	15	9.1	7.5	7.4	11.6
Platelet count	10 × 3/μL	253	229	181	232	254
Hemoglobin	g/dL	17.8	16.4	15.5	14.3	12
Blood culture	—	No growth	—	—	—	—
C‐reactive protein quantitative	mg/L	1	1	9.6	2.8	
Aspartate aminotransferase	U/L	—	43	39	—	—
Alanine transaminase	U/L	—	19	18	—	—
Bilirubin, total	mg/dL	—	5.8	1.9	0.6	—
Bilirubin, direct	mg/dL	—	0.3	0.5	—	—
Creatinine, serum	mg/dL	—	0.46	0.46	0.43	0.43
Blood urea nitrogen	mg/dL	—	6	3	5	22
Calcium, serum	mg/dL	—	8.6	8.5	8.6	—
Magnesium, serum	mg/dL	—	1.64	—	—	—
Sodium, serum		—	137	144	145	140
Chloride, serum		—	109	115	109	105
Potassium, serum		—	3.5	4.6	4.5	4.3

**FIGURE 2:**
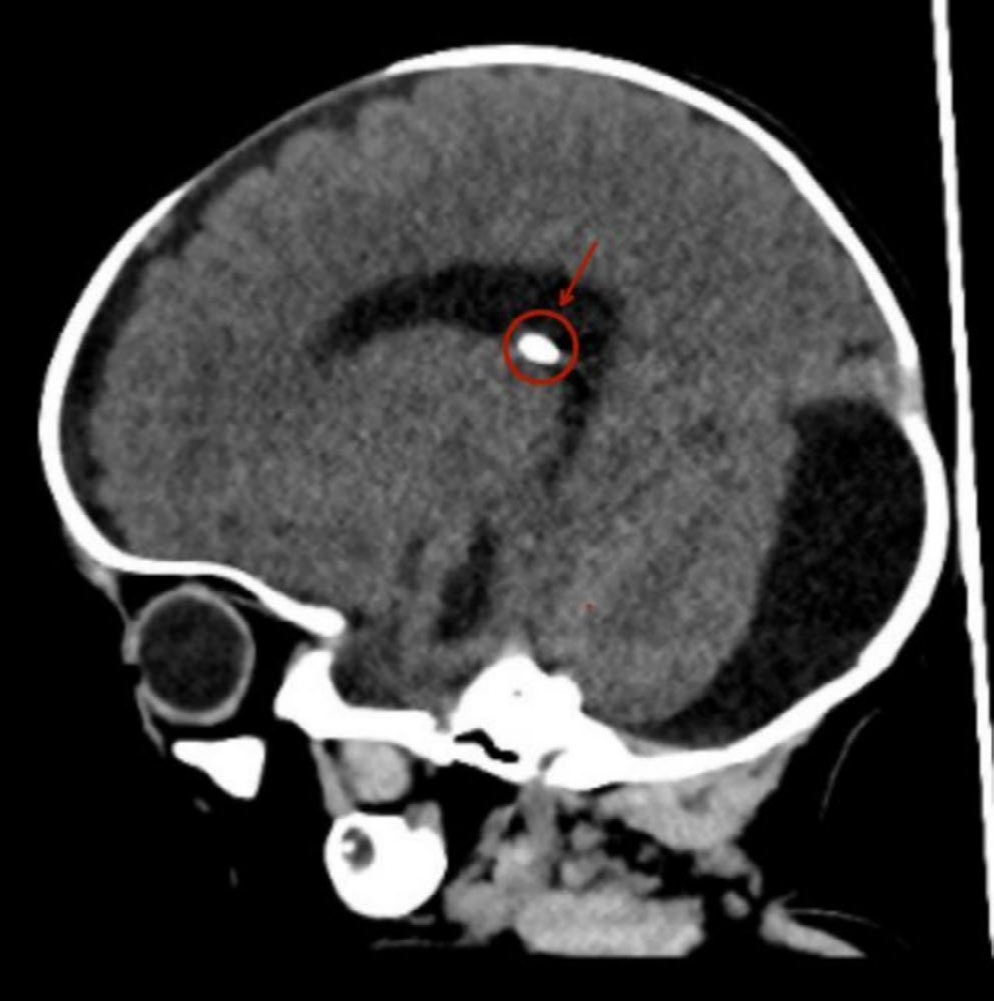
CT Scan of the brain. Ventricular drainage device, shown in red, seen within the lateral ventricle.

A month later, Due to persistent elevation of intracranial pressure, a right parietal ventriculoperitoneal (VP) shunt was placed to relieve the obstruction (Figure 2).

## 4 Outcome and Follow‐Up

At the age of five months, the infant exhibited gradual clinical improvement with no recurrence of seizures. His respiratory status stabilized, allowing for the discontinuation of supplemental oxygen, and routine laboratory tests during this period remained unremarkable (Table 1). Neurologically, the VP shunt placement alleviated the signs of raised intracranial pressure, resulting in improved alertness and feeding.

In addition, the family was counseled extensively regarding the infant's condition, including discussions about potential genetic implications, further reinforcing the significance of the negative family history.

Developmental follow‐up with pediatric neurology remains ongoing to monitor for potential motor or cognitive delays associated with DWM. In parallel, cardiac follow‐up is planned to track the VSD and PFO, though both defects continue to be hemodynamically insignificant. The family was provided with genetic counseling and comprehensive education about the infant's condition, emphasizing the importance of long‐term multidisciplinary care.

## 5 Discussion

The case presentation of this 5‐month‐old male with DWM, a rare autosomal dominant congenital malformation of the brain in which there is incomplete development or absence of the cerebellar vermis, with cystic enlargement of the fourth ventricle and the presence of a cyst in the posterior fossa [7]. This case was typical, as described in other reported cases of this congenital disorder. Findings present a wide spectrum of clinical manifestations, ranging from very mild to severe presentations [8]. In this case, it includes macrocephaly, hypotonia, respiratory distress, and suspected seizures—all common manifestations of the condition, though presentations may vary among patients. Macrocephaly, defined as a head circumference above the 95th percentile, is commonly cited in cases of DWM resulting from ventriculomegaly and hydrocephalus due to impaired CSF flow. This is further supported by research studies by Epelman et al. and Phillips et al. [9, 10], since macrocephaly is an often‐recurring characteristic that raises neurological awareness of the necessity of early intervention to decompress intracranial pressure.

Neurological features consist of hypotonia and diminished reflexes, including a weak Moro and sucking reflex, consistent with the defects in motor function and coordination resulting from DWM cerebellar vermis agenesis [11]. Literature in Radiology and other studies has also presented neurological deficiencies in affected infants, which include considerable motor and cognitive delays, although the outcomes are varied depending on the extent of cerebellar malformation [12]. Seizures can occur in DWM cases, though they are rare—as seen in this case—and may be linked to impaired CSF dynamics or other congenital brain anomalies [13, 14].

The respiratory distress, shortly after birth in this case, might be related to increased intracranial pressure or to other central nervous system involvement that complicates the presentation of DWM in neonates [15]. Indeed, other studies, such as Chang et al., report that respiratory compromise, while not universally present, is observed in some infants with DWM—especially those with significant hydrocephalus [16, 17]. NICU support, as provided in this case, is imperative for stabilizing respiratory function in these situations.

The DWM shows a strong association with other congenital anomalies [12]. These include a small muscular VSD and PFO in this patient. The strong association of DWM with other congenital defects has been emphasized in various studies, especially cardiac anomalies like VSDs and ASDs [18]. As indicated in the WHO EMRO report, among others, these cardiac anomalies, though mostly hemodynamically insignificant at the outset, suggest a benefit for routine follow‐up, as they may influence long‐term cardiovascular health [19]. Again, this multisystem involvement calls for a unified approach to care in DWM cases, since the spectrum of related anomalies may affect short‐ and long‐term outcomes.

Imaging studies are very important for diagnosis. DWM is incidentally diagnosed on routine prenatal ultrasound, which includes cystic enlargement of the fourth ventricle with absence or hypoplasia of the vermis as shown in this case [20]. However, in cases of late diagnosis at birth or even after birth, MRI is the modality of choice, as it provides detailed visualization of the brain's anatomy and confirms the typical features seen in DWM [21]. However, if an MRI is unavailable, a CT scan can help confirm the diagnosis as in this case [22].

The findings in this case of DWM—including an absent cerebellar vermis, cystic dilation of the fourth ventricle, and dilated lateral ventricles—closely align with the features most commonly associated with typical DWM, as reported in several studies (Table 2) [10, 23]. These structural defects often result in hydrocephalus, as also nearly seen in this infant, which might need intervention as a way of dealing with the raised intracranial pressure. Imaging studies are very important for the diagnosis of DWM [16]. Classically described features include cystic dilation of the posterior fossa, vermian hypoplasia, and enlargement of the fourth ventricle. Various studies from Children's National Hospital describe manifestations not very unlike these findings and further emphasize early imaging to assess the severity and guide intervention [24, 25].

**TABLE 2:** provides a comparative overview of the key clinical characteristics, associated anomalies, imaging findings, management, and outcomes in our case and related cases reviewed in the literature.

Study/source	Key symptoms & presentation	Congenital anomalies	Imaging findings	Management & outcome
Current case	Macrocephaly, hypotonia, respiratory distress, seizures	Small muscular VSD, PFO	Cystic dilation of the fourth ventricle, absent cerebellar vermis, ventriculomegaly	VP shunt placement for raised ICP, multidisciplinary follow‐up
Chang et. al	Hydrocephalus, hypotonia, seizures	High incidence of cardiac defects (VSD, PFO)	Cystic dilation of the fourth ventricle, absent cerebellar vermis	VP shunt improves pressure; developmental delays common
Epelman et. al	Macrocephaly, delayed milestones, psychogenic changes	Cardiac and occasional renal anomalies	Posterior fossa cysts, hydrocephalus	High success with dual shunt; single shunt has risk of herniation
Al‐Turkistani et. al	Seizures, hypotonia, respiratory distress at birth	Increased incidence of non‐CNS malformations	Consistent with DWM spectrum	VP shunt favored over excision due to lower complication rates

The use of VP shunts in the relief of hydrocephalus among cases of DWM is well supported. An example is that a study from the Journal of Neurosurgery indicated that the presence of hydrocephalus, present in approximately 73% of patients with DWM, was a major predictor of outcomes, with VP shunting offering immense relief in symptoms by aiding in preventing further neurological complications [26]. Long‐term results vary, but it would appear that in most patients, shunting improves feeding, motor responses, and cognitive functioning. On the other hand, a small population of patients might still suffer from motor and cognitive delays depending on other anomalies in the CNS or the severity of hydrocephalus [25].

Further reports suggest that such a dual‐shunt system, including things like cyst‐peritoneal and ventriculo‐peritoneal shunts, for example, is far more effective in treating both the supratentorial and infratentorial components of hydrocephalus [27]. The mainstay of treatment has remained single VP shunts owing to relatively lower complication rates and better symptom management, as reflected in the presented outcomes from the WHO EMRO study and Cleveland Clinic [28, 29]. Follow‐ups are always required, since though rare, complications such as shunt blockage or infection can affect the new developmental endpoint if intervention is not done in time (Table 2).

Developmental outcomes may vary widely in infants with DWM and depend significantly on the extent of cerebellar and ventricular involvement, presence of hydrocephalus, and associated CNS anomalies. Therefore, in this case, continued follow‐up for motor and cognitive development is appropriate with established recommendations for multidisciplinary care to address potential developmental delays associated with DWM.

Studies have identified that, with DWM, children often have delays in learning to sit and walk; mental development and language are occasionally delayed [30, 31]. Research at Children's National Hospital has indicated that poor coordination and balance—common in DWM—are often related to malformations of the cerebellum [32]. The degree of impairment may vary depending on the severity of the structural abnormalities [33]. These delays are usually indicative of early interventions, physical therapy, occupational therapy, and speech therapy to support the acquisition and adaptation of skills.

One study, reported in the Journal of Neurosurgery: Pediatrics, followed children with DWM through most developmental stages and found that results were better for children with mild varieties of DWM without other CNS abnormalities (Table 2) [26]. Children with significant hydrocephalus or other structural brain abnormalities, such as agenesis of the corpus callosum, had more significant delays in motor and cognitive areas [34, 35]. This variability brings out the importance of an individualized developmental follow‐up plan, long‐term, which has already been started by this team in monitoring the various developmental milestones and instituting early interventions, as necessary.

To enhance developmental outcomes in children with DWM, a multidisciplinary approach is widely recommended. Studies highlight the importance of regular assessments by pediatric neurologists, developmental specialists, and therapists to address motor and cognitive challenges [6]. Research indicates that individualized education plans (IEPs) and consistent therapies, such as physical, occupational, and speech therapy, are beneficial for children with DWM, especially when intellectual delays or motor impairments are present. In this case, the follow‐up plan involving neurology and developmental specialists aligns with these recommendations, ensuring early intervention and timely support to manage developmental needs as they arise [36].

Furthermore, early detection of DWM is critical not only for immediate management but also for anticipating potential long‐term neuropsychiatric sequelae. Although many cases remain asymptomatic during infancy, subtle neurodevelopmental impairments may emerge later in adolescence and young adulthood. For instance, insights from the systematic review by Bortoletto et al. (2024) highlight that patients with DWM may develop psychiatric profiles, including tic‐related obsessive‐compulsive and eating disorders, which underscore the importance of early detection and ongoing neuropsychiatric evaluation [37].

Genetic counseling forms an integral part of the management of DWM cases since the condition has a real potential for genetic underpinning. Indeed, from studies, it is seen that DWM may sometimes present with chromosomal abnormalities such as trisomies 13 and 18, or even specific genetic syndromes like Walker‐Warburg and Meckel‐Gruber [6]. These associations suggest a genetic basis for recurrence risks, which are estimated to be between 1% and 5% in the case of isolated DWM but higher if related to specific syndromic patterns. Therefore, genetic counseling may be informative for the family with respect to recurrence risk in subsequent pregnancies and decisions regarding prenatal testing, including karyotyping or genetic sequencing, for early detection of anomalies (Table 2) [38].

Genetic counseling informs families about potential developmental and neurological outcomes associated with DWM. Research has shown that genetic counseling enables families to anticipate a broad range of developmental outcomes since intellectual and motor impairments in children with DWM may considerably vary [39]. It is believed that such anticipation helps families plan accordingly, with early intervention strategies comprising essential parts of multi‐professional treatment to optimize developmental outcomes.

By aligning the expectations of the family and through the repetition of knowledge regarding the implications of the genetic link to DWM, genetic counseling also supports making informed decisions that allow families to be aware of recurrence risks and long‐term management strategies that may offer improvements in the quality of life for affected children. Such an approach is supported at multiple literature levels within genetics and pediatrics and recommendations from fetal medicine specialists themselves.

## Author Contributions

**Mohammad Bdair:** conceptualization, supervision, validation, visualization, writing – original draft, writing – review and editing. **Fathi Milhem:** conceptualization, resources, supervision, validation, writing – original draft, writing – review and editing. **Zaid Sawaftah:** validation, visualization, writing – original draft, writing – review and editing. **Husam Hamshary:** data curation, writing – original draft, writing – review and editing. **Hamza A. Abdul‐Hafez:** validation, writing – original draft, writing – review and editing. **Moath Hattab:** validation, writing – original draft, writing – review and editing. **Noor Nabresi:** validation, writing – original draft, writing – review and editing. **Omar Naseef:** validation, writing – original draft, writing – review and editing.

## Ethics Statement

Our institution does not require ethical approval for reporting individual cases. This study was performed in accordance with the Helsinki Declaration of 1964 and its later amendments.

## Consent

Written informed consent was obtained from the patient for their anonymized information to publish this report in accordance with the journal's patient consent policy.

## Conflicts of Interest

The authors declare no conflicts of interest.

## Data Availability

Data sharing not applicable to this article as no datasets were generated or analysed during the current study.
